# Monitoring improvements in the nutritional quality of new packaged foods launched between 2016 and 2020

**DOI:** 10.3389/fnut.2022.983940

**Published:** 2022-11-01

**Authors:** Marie Tassy, Andréas Rytz, Adam Drewnowski, Alec Lecat, Emma F. Jacquier, Véronique Rheiner Charles

**Affiliations:** ^1^Nestlé Research, Vers-chez-les-Blanc, Lausanne, Switzerland; ^2^Department of Epidemiology, Center for Public Health Nutrition, School of Public Health, University of Washington, Seattle, WA, United States

**Keywords:** Mintel Global New Products Database, nutrient profiling, food supply, packaged food, monitoring

## Abstract

Food and beverage companies reformulate packaged foods and to better align their products with public health policies and evolving consumer needs. The nutritional quality of packaged foodscan be tracked using nutrient profiling methods. The present study compared the nutritional quality of packaged foods launched globally between 2016 and 2018 and those launched in 2018–2020, as reported in the Mintel Global New Products Database. Nutrient profiling analyses showed that the nutrient composition of product categories shifted for almost 40% of newly launched products between 2016–2018 and 2018–2020. For example, pizzas that had been assigned to one nutritionally homogenous category in 2016–2018 separated in two nutritionally distinct subgroups in 2018–2020. The new products that were reduced in protein, saturated fat, and sodium were now nutritionally distinct from the traditional pizza offer. By 2018–2020 “best of category” products were significantly lower in sugar and sodium than before; however, no clear trend was observed for saturated fats, proteins, or fibers. The relative speed of product reformulation was category specific. This analysis of the Mintel Global New Products Database suggests that the WHO nutritional recommendations focusing on sugar and sodium reduction did have a positive impact on the composition of new packaged foods, whether through reformulation of existing products, launches of line extensions or new product development.

## Introduction

The World Health Organization has urged food companies to lower the fat, sugar, and salt content of packaged foods (1), but it is not easy to evaluate the global impact of these recommendations on the evolution of the nutritional quality of packaged foods. This impact can be tracked using the Mintel Global New Products database, which lists, annually, more than 130,000 newly launched products in 86 countries along with their energy and nutrient content. Mintel product categories are linked to product manufacturing and marketing and are commonly used by food and beverage industries because they provide an actionable and up-to-date representation of the global food supply (2).

The present objective was to monitor the changes in the nutrient content of the global food supply, as tracked by Mintel, during two time periods: 2016–2018 and 2018–2020. The present method was based on nutrient profiling of product categories. Food manufacturers use nutrient profiling models that rank or classify foods based on their energy and nutrient content to continuously reformulate existing products and to set nutrition targets for line extensions and product innovation (3). While many nutrient profiling models are across-the-board (4), food manufacturers prefer to use nutrient profiling models that are category-specific and better correspond to product lines (5). One problem with those food category assignments that are driven by marketing or product positioning is that they can include foods that are very heterogeneous from the nutrition standpoint. Breakfast cereals or pizza can be of variable nutrient density. To overcome this problem, we have developed a new methodology (6) to disaggregate marketing categories into product subgroups or “classes” that were more nutritionally homogeneous. That procedure was used to set reformulation targets for new products that were comparable to those that in 2016-18 were judged to be “best of class” (6).

Analyses of emerging packaged foods can inform reformulation and innovation efforts by the food industry. First, monitoring nutrient composition of new product launches is one way to identify the most common reformulation targets. Such analyses can provide additional insights as to the emergence of new product categories that are distinct from the more traditional products on offer. Second, given the global nature of Mintel data, such analyses can help determine whether product reformulation is occurring globally and whether it affects multiple product categories. Third, the present study explored whether consumers were effectively informed about the observed evolutions through changes on a category specific front-of-pack labeling system.

## Materials and methods

The present model relies on three algorithms to analyze the distributions of five public health sensitive nutrients—total sugars, total sodium, and saturated fat along with protein and fiber (7)—that were recently developed and discussed in detail (6). The first algorithm identified which of the five considered nutrients were relevant for each category; a nutrient was considered category-relevant if more than half of the products within this category had a declared nutrient content with a value that exceeded the “low in” or the “source of” limit. The second algorithm identified nutritionally homogeneous subcategories by analyzing the distribution modes of the category relevant nutrients. The third algorithm used a multivariate percentile approach to derive subcategory specific nutrient targets.

The Mintel Global New Products Database for the period 2016–2018, lists 416,706 packaged foods. Based on their nutrient content, products with relevant labeled information can be analyzed and portioned into 292 nutritional homogeneous subcategories. For the full 3-year period 2018–2020, the Mintel Global New Products Database lists 397,958 products that can be split into 288 nutritional subcategories. In a previous study, we used 350,994 products for 263 subcategories, but the 3 years period was truncated to 30 months instead of 36 (6).

Data for these two 3-year periods—with a 1-year overlap—were analyzed to identify both the structural changes of the categorization and the nutritional changes within categories. Since both sugars and sodium have been targeted by global reduction campaigns around the world (8), special attention was given to categories such as yogurts that significantly contribute to sugar intakes (9), as well as noodles and pizzas that are highly consumed all around the world (10) and that significantly contribute to sodium intakes (11, 12). Bread and cheese were among categories featuring large numbers of launched products.

Additional algorithms were developed to assess the evolution of both the food supply category structure and the targets within nutritionally homogenous subcategories. These algorithms are introduced through examples to make them most intuitive.

### Partition-matching algorithm to assess the structural evolution of nutritional categories

When applying the model on both considered periods, it appears that Mintel categories such as “Bread and Bread Products” or “Hard Cheese and Semi-Hard Cheese” do not require any further subcategorization. Although featuring products that cover a wide range in terms of sodium and protein, their distributions are unimodal and therefore reveal that they belong to one homogeneous nutritional category. For such cases, the structure remains stable.

Similarly, the Mintel category “Pizzas” is nutritionally homogeneous in 2016–2018 but the protein distribution appears to become bimodal in 2018–2020, indicating that two nutritionally different pizza subcategories have emerged. On the contrary, the Mintel category “Plant Based Spoonable Yogurts” moved from two sugar modes in 2016–2018 to one homogeneous category in 2018–2020. These examples are simple cases of structural evolution with only one category in either one of the two periods considered for the evolution.

For Mintel categories such as “Spoonable yogurts” and “Instant Noodles” with, respectively, two and four nutritionally homogeneous subcategories in both 2016–2018 and 2018–2020, the structural evolution can only be assessed if being able to match the two partitions of subcategories. For doing so, since the two periods were overlapping 1 year, it was possible to develop a simple partition matching algorithm. To illustrate this algorithm, the example of “Instant Noodles” is displayed (Table 1). As a first (A), the number of products launched in 2018—the year that is common to the two considered periods—is cross-tabulated across the two partitions. As a second (B), the proportional distribution of each old category (2016-18) across the new categories (2018–2020) is calculated. Finally, the algorithm matches any new category with the old category for which its prevalence was highest. In this example, New.1 is matched with Old.1, because prevalence of New.1 was 84% in Old.1 and 0% elsewhere; New.2 is matched to Old.2, because prevalence of New.2 was 35% in Old.2 and lower elsewhere; similarly New.3 is matched with Old.3 and New.4 is matched with Old.4. If the prevalence for a new category was the same in two old categories, the new category was matched with the larger of the two old categories. This method ensures that each new category is matched to a unique old category. In cases an old category is not matched with a new category, the algorithm is simply reversed.

**TABLE 1:** Total of 1,476 products in Mintel category “Instant Noodles” in 2018.

	New.1	New.2	New.3	New.4	New.Sum
**(A)**					
Old.1	228	27	13	4	272
Old.2	0	159	0	292	451
Old.3	0	0	27	6	33
Old.4	0	0	0	720	720
Old.Sum	228	186	40	1,022	1,476
**(B)**					
Old.1	84	10	5	1	100
Old.2	0	35	0	65	100
Old.3	0	0	82	18	100
Old.4	0	0	0	100	100

Split into four nutritionally distinctive subcategories for both periods 2016–2018 (Old) and 2018–2020 (New) with (A) cross-tabulation of the two partitions and (B) Proportional distribution of each old category across all new categories. Underlined values represent the old category in which prevalence of new category is the highest.

### Weighted-average algorithm to assess the evolution of nutrient quality

For all nutritionally homogenous subcategories that can be matched one to one over the two periods, the comparison of thresholds between the two periods is straightforward. It can be expressed as a relative improvement for reduction of disqualifying nutrients and increase of qualifying nutrients. Results focus on relative changes larger than 5% to account for the evolutions having highest nutritional impact. This threshold of 5% could naturally be adapted to match alternative objectives, but due to the limited number of products in certain categories, smaller changes should be considered with caution.

For a case like pizza that matches one subcategory of 2016–2018 with two subcategories of 2018–2020, the comparison of thresholds is performed after averaging the thresholds of the two categories using a weighted average approach, with the weights given by the number of products in each of the two subcategories (Table 2).

**TABLE 2:** Total of 4,025 products in Mintel category “Pizzas” for the period 2018-20, with nutritional thresholds for the two nutritionally homogenous subcategories and their weighted average (e.g., Mean Sodium threshold = 592 mg/100 g = (572*2,831 + 640*1,194)/4,025), and comparison with the thresholds of the 3928 pizzas of 2016–18.

	N	Sfa (g/100 g)	Sodium (mg/100 g)	Protein (g/100 g)
Pizzas 2018–2020 (1)	2,831	4.6	572	8.0
Pizzas 2018–2020 (2)	1,194	6.2	640	11.6
Pizzas 2018–2020 (weight. Av.)	4,025	5.1	592	9.1
Pizzas 2016–2018	3928	5.0	600	8.8
Relative improvement		−2%	+1%	+3%

### Assessing health star rating evolution within Mintel categories

To compare the launches of 2016–2018 with those of 2018–2020 in a more direct and holistic way, the Health Star Rating (HSR) of all launched products was estimated (13). Since this estimation relies on complete labels, the estimate could only be done for less than 90% of all products considered previously.

Among other widely recognized front-of-pack labeling systems, HSR was selected because it was designed to enable an easy and standardized comparison of packaged foods. It differentiates nutritional quality within and between categories, is publicly available and gives a holistic view of the nutritional quality of the product. It is based on a scoring algorithm that deducts points for disqualifying nutrients (overall energy, sodium, total sugar, and saturated fat) and adds points for qualifying nutrients and ingredients (protein, fiber, and fruit and vegetables). These scores are converted to a “Health Star Rating” between 1/2 to 5 stars. This rating scale featuring 10 levels allows to discriminate products more than other systems that rely on less levels. HSR has therefore been chosen to increase the chances to observe changes, but any other front-of-pack labeling system could have been used.

The average HSR ratings of each Mintel category was calculated and compared between the two periods of time.

## Results

### Structural evolution of nutritional categories between 2016 and 2020

**FIGURE 1:**
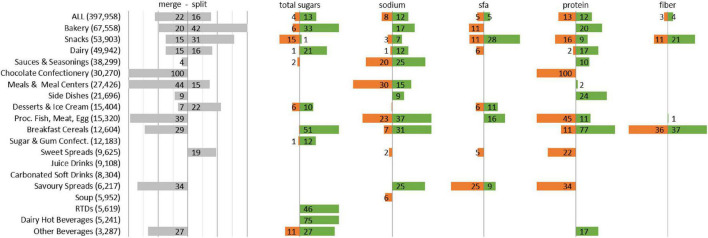
Percentage of products belonging to a nutritionally homogeneous subcategory in 2018–2020 that either merged two or more subcategories of 2016–2018 or that split from a larger category. For each nutrient, percentage of products belonging to a subcategory for which the nutrient threshold improved by at least 5% (green) or was relaxed by at least 5% (orange) between 2016–2018 and 2018–2020.

The partition matching algorithm yields three cases: (1) a single subcategory in 2016-18 matching a unique one in 2018–2020, (2) two or more subcategories merging into one, and (3) a single subcategory splitting into two or more smaller ones (Figure 1).

As a result, 62% products launched in 2018–2020 belong to a subcategory that remained stable between 2016–2018. The structure of the global Mintel categories “Sugar and Gum Confectionery,” “Juice Drinks,” “Carbonated Soft Drinks,” “Soup,” “RTDs,” and “Dairy Hot Beverages” remained completely stable.

Moreover, 22% products launched in 2018–2020 belong to a subcategory that merged two or more subcategories since 2016–2018, indicating that the competitive landscape became more homogeneous in terms of nutritional quality; this tendency is strongest, with more than 40%, for the three global Mintel categories “Chocolate Confectionery,” “Meals and Meals Centers,” and “Processed Fish, Meat and Egg.”

Finally, 16% products launched in 2018–2020 belong to a subcategory that split from a subcategory that was larger 2 years before. This tendency is strongest, with more than 20%, for the three global Mintel categories “Bakery,” “Snacks,” and “Desserts and Ice-Cream.”

### Evolution of nutrient thresholds within subcategories between 2016 and 2020

Between 2016 and 2020, 83% of all products belong to subcategories for which the total sugars threshold did not evolve by more than 5%. It improved for 13% and was relaxed in only 4%, leading to a positive difference of 9% (Figure 1). This difference is + 75% in “Dairy Hot Beverages,” + 51% in “Breakfast Cereals,” + 46% in “RTDs,” + 27% in “Bakery,” + 21% in “Dairy,” + 16% in “Other Beverages” and + 11% in “Sugar and Gum Confectionery.” The difference is negative for “Snacks” (-14%). No evolution is observed for categories such as “Meals and Meal Centers,” “Side Dishes,” “Processed Fish, Meat and Egg” or “Soups” for which sugar is not a category-relevant nutrient.

For sodium, the threshold improved for 12% and was relaxed in 8%, leading to a positive difference of + 4%. This difference is + 25% in “Savory Spreads,” +24% in “Breakfast Cereals,” + 17% in “Bakery,” + 11% in “Dairy,” + 9% in “Side Dishes” and + 5% in “Sauces and Seasonings.” The difference is negative for “Meals and Meal Centers” (-15%). However, 15% products being assessed vs. stricter thresholds are products with an average of more than 1300 mg/100 g, whereas the 30% of food products being assessed vs. more relaxed thresholds are products with an average of less than 430 mg/100 g.

For saturated fat, protein and fibers, no global overall trend is observed, but in some categories, interesting patterns are observed with noticeable efforts to reduce saturated fat in “Snacks” and “Processed Fish, Meat and Egg” or to increase proteins in categories such as “Breakfast Cereals,” “Side Dishes,” “Bakery,” “Other Beverages” and “Dairy” or fibers in “Snacks.”

### Evolution of structure and nutrient threshold in selected categories

**FIGURE 2:**
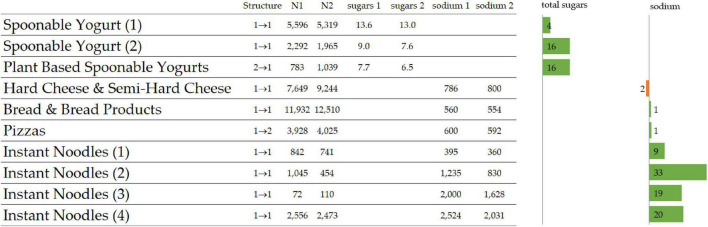
Ten selected nutritionally homogeneous subcategories, type of structural changes between 2016 and 2020, Number of products N1 for 2016–2018 and N2 for 2018–2020, sugar content (g/100 g) and sodium content (mg/100 g) and relative improvement between the two periods as bar charts.

Results for selected categories focus on total sugars and sodium because no noticeable evolution could be observed for the other nutrients in these categories (Figure 2). The Mintel category “Spoonable Yogurts” featured two nutritionally homogenous subcategories both in 2016–2018 and 2018–2020: the first with higher thresholds in total sugars and saturated fat than the other. An improvement was observed for both subcategories with a decrease of the total sugar threshold of 4% for the first and of 16% for the second subcategory moving from 9.0 to 7.6 g/100 g. The two subcategories that constituted “Plant Based Spoonable Yogurts” merged into one homogenous category with a total sugar threshold that was decreased from 7.7 to 6.5 g/100 g (16% improvement).

No significant evolution in structure or quality was observed for “Hard Cheese and Semi-Hard Cheese” and “Bread and Bread Products.” The single Pizzas category of 2016–2018 split into two subcategories in 2018–2020 (Table 2). Indeed, the distribution of protein content of Pizzas category which was unimodal in 2016–2018 and ranged from 0 g/100 g to 23.1 g/100 g, became bimodal in 2018–2020 with one mode at 9.8 g/100 g and one at 12.4 g/100 g, and a split between the two modes at 11.1 g/100 g. The two subcategories of 2018–2020 have significantly different content of protein (45%), saturated fat (35%), and sodium (12%). However, when comparing globally the “pizza” category between the two time periods, none of these nutrients changed by more than 5%.

For “Instant Noodles,” the structure did not change with four nutritionally homogenous subcategories. Interestingly, for all four subcategories, the sodium threshold improved between 9% (for low sodium noodles) to 19–33% for the three other subcategories (higher sodium).

### Evolution of health star rating in selected categories

**FIGURE 3:**
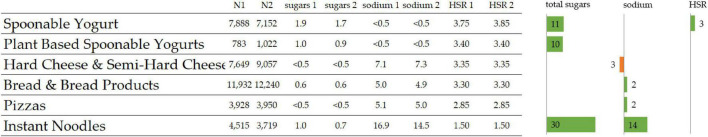
Six selected Mintel categories, Number of products N1 for 2016–2018 and N2 for 2018–2020, mean HSR points for total sugar and sodium, mean HSR, and relative improvement between the two periods as bar charts.

Overall, HSR scores and ratings could be estimated for less than 90% of products, due to incomplete nutritional declaration. For the previously selected categories, there was no such issue since HSR could be estimated for more than 98% products. Overall, the findings on total sugar and sodium improvements derived from HSR ratings are well aligned with those derived from the threshold method (Figure 3).

For total sugars, mean HSR scores show that total sugars have decreased by respectively 10 and 11% for “Spoonable Yogurts” and “Plant Based Yogurts.” Furthermore, this decrease was even 30% for “Instant Noodles”; this latter decrease was not identified by the threshold method, simply because total sugars is not a category-relevant nutrient.

For sodium, the only significant improvement in mean HSR scores is observed for “Instant Noodles” with a 14% decrease. This is overall aligned with the mean evolution of the thresholds but lacks the granularity that could be observed for the different “Instant Noodle” subcategories.

Finally, in terms of mean HSR rating, no significant evolution could be observed. Mean HSR rating remained stable with 3.4 stars for “Plant Based Spoonable Yogurts,” 3.35 stars for “Hard Cheese and Semi-Hard Cheese,” 3.30 stars for “Bread and Bread Products,” 2.85 stars for “Pizzas” and 1.50 for “Instant Noodles.” For “Spoonable Yogurt,” the 11% decrease in total sugars was sufficient to yield a 3% increase in the mean HSR rating, moving from 3.75 to 3.85 stars. At the other end, “Instant Noodles,” although decreasing sodium by 14% was not yielding an increased mean HSR rating.

## Discussion

The present analyses confirm the effect of WHO nutritional recommendations to reduce sugar and sodium in packaged foods. Compared to year 2016, by 2020, the “best of class” products were overall significantly lower in both sugar and sodium. By contrast, no clear trend could be observed for the reduction in saturated fats, or for any increase in protein, or fiber content of food. Those too are among the targets for product reformulation by the food industry. To our knowledge this work is the first one to monitor the changing nutrient composition of the global packaged food supply. Many other studies have either focused on specific countries (14) or single nutrients such as sugar (15) or sodium (16).

Further, product categories identified in 2016 were not the same as product categories identified in 2020. The nutrient content of foods in nutritionally homogeneous categories evolved for more than one third of the products. Each of the 292 nutritionally homogeneous categories of 2016–2018 could be matched with one of the 288 categories of 2018–2020. This matching showed that between 2016 and 2020, both the category structure and the quality within categories evolved noticeably. Thus, the profiling system previously developed to effectively guide product reformulation enables one to determine category-specific nutrient thresholds applicable at one time point, and, when combined with a partition matching algorithm, to monitor the qualitative and quantitative changes in the manufactured food supply over the years.

Monitoring new product launches can be used to track the appearance, or disappearance, of food products on offer and to assess their nutritional quality. The Pizza category that was unique and homogeneous in 2016–2018 evolved into two subcategories with distinctive nutrient profiles between 2018 and 2020. The two categories were identified through a bimodal distribution of protein, but their content of saturated fat and sodium were also significantly different. This is because meat and cheese content of pizzas simultaneously affect protein, sodium and saturated fat. The newly emerging pizza category contained less nutrients of public health concern, reflecting perhaps current trends toward improved nutrition. Since Mintel does not report the volumes or sales of new launches, how consumers reacted to the changes in products offering is not known. Further study could aim looking at evolution of packaged food supply on a longer time, to detect lasting trends in product food offering which have been accepted by consumers.

The new pizza category may have been the result of reformulation or new product entries (17). Emerging new categories were also observed for “Bakery,” “Snacks,” and “Desserts and Ice-Cream,” suggesting that those categories were also targets for reformulation and for improving nutrient density. On the other hand, Plant Based Spoonable Yogurts were split into two categories in 2016–2018, based on sugar content. Two years later, Plant Based Spoonable Yogurts became a single category with a sugar content that was uniformly low. For confectionery and snacks, tracking shifts in category membership is especially difficult. Such products can contain multiple ingredients and can be of highly variable nutritional value, making it difficult to detect changes over time.

For the most part, product categories remained stable between 2016 and 2020. New product categories were characterized by less total sugars and less sodium. Both dairy yogurts and their plant-based alternatives’ sugar thresholds decreased by 15%, a trend observed for almost one fourth of dairy products, and sodium thresholds dropped for 12% of 2018–2020 new launches. A cut in sodium and sugars was observed mostly in categories where levels were high, respectively, more than 1350 mg/100 g and 20 g/100 g, showing that reduction efforts focus on the subcategories where it matters most, while not stopping the effort even in the more healthful subcategories. This analysis is therefore reflecting the consequences of major WHO nutritional policies of the past decade (18) and its implementation by countries (19, 20) and efforts by food manufacturers (21–24) who focused on categories were the contents were critical. The changes for unsaturated fat, protein and fiber thresholds are less obvious when looking at global new launches, however, significant changes happened at category levels. More than 25% of dairy products were assessed against stricter protein thresholds after 2 years, and more specifically soft, semi-soft and processed cheese, which may reflect the recent trends for low fat and high protein products (25) and consumers looking for help with weight management (26).

Nutrient profiling of product categories provides a more precise way to track changes in the global food supply when compared to regional front-of-pack labeling system such as HSR. Indeed, both methods showed a decrease in the sodium content of Instant Noodles, and an absence of significant variation in the nutritional quality of pizzas after 2 years. Yet there was no improvements in HSR ratings. Moreover, the HSR analysis did not catch the reduction of sugar threshold of low sugar Spoonable Yogurts. The analysis herein demonstrates an alternative approach to detect nutritional changes in packaged food, which HSR might miss. This type of analysis may be useful for future application to demonstrate, comprehensively, the nutritional evolution of the food supply.

## Conclusion

The present analysis of the Mintel Global New Product Database shows that the nutrient content of packaged foods significantly improved over a 5-year period (2016–2020). As trajectories of change were category specific, targets for reformulation should be periodically revisited to promote a continuous improvement of the global packaged food supply. Further analyses could apply this methodology to other product nutrient composition databases and could include volumes and sales data to assess, more accurately, the impact of WHO recommendations and consumer trends on global consumption.

## Data availability statement

The data analyzed in this study is subject to the following licenses/restrictions: Data was obtained from 2020 Mintel Group Ltd. Requests to access these datasets should be directed to https://www.mintel.com/global-new-products-database.

## Author contributions

MT, AR, and VC: conceptualization, formal analysis, methodology, and writing—original draft. AD, EJ, and AL: supervision and writing—review and editing. All authors have read and agreed to the published version of the manuscript.
